# Macular degeneration and occupational risk factors: a systematic review

**DOI:** 10.1007/s00420-018-1355-y

**Published:** 2018-09-06

**Authors:** Alberto Modenese, Fabriziomaria Gobba

**Affiliations:** Department of Biomedical, Metabolic and Neural Sciences, University of Modena and Reggio Emilia, Via G. Campi 287, 41125 Modena, Italy

**Keywords:** Macular degeneration, Occupational exposure, Solar radiation, Blue light, Ultraviolet radiation, Chemical exposure

## Abstract

**Purpose:**

Macular degeneration is a multi-factorial disease, leading cause of blindness for people over 50 years old in developed countries. To date, the knowledge on possible occupational factors involved in the development of the disease is scant.

**Method:**

We performed a systematic scientific literature search on the association between macular degeneration and occupational risk factors searching the MedLine and Scopus databases.

**Results:**

We examined 158 articles and, according to the inclusion criteria, 13 peer-reviewed studies evaluating occupational risk factors for macular degeneration or reporting the frequency of the disease in specific groups of workers were included in the review. Ten on thirteen articles evaluated the presence of macular degeneration in workers exposed to solar radiation. Only one study found that non-specific history of occupational chemical exposure was associated with the disease. Two studies showed an association between macular degeneration and the general category of “blue-collar” workers, but they did not identify the specific risk factors involved.

**Conclusions:**

To date few studies have examined occupational risk factors for macular degeneration. Nevertheless, available data indicate that long-term occupational solar radiation exposure, in particular for its blue-light component, is associated with macular degeneration in outdoor workers.

**Electronic supplementary material:**

The online version of this article (10.1007/s00420-018-1355-y) contains supplementary material, which is available to authorized users.

## Introduction

Macular degeneration (MD) is a chronic eye disease affecting the macula: the progressive loss of vision typical of the MD, mainly in the centrum of the visual field, has a quite slow evolution and it can take years after the first diagnosis to induce an appreciable visual impairment (Christoforidis et al. 2011; Evans 2001; Shah et al. 2007). MD is currently the leading cause of blindness for people over 50 years old in developed countries (Taylor et al. 2001). Its prevalence in Europe is 3.3% (Augood et al. 2006), and it is similar also in the US, where about 10 million of people are affected (Tomany et al. 2004a, b).

There are two major forms of MD, with different prognosis and therapy: the atrophic or dry-type MD, representing approximately the 85–90% of the total number of cases, and the neovascular or wet-type MD. The initial alteration of the fundus is often represented by the so-called drusen, or colloids bodies, that are degenerative formations of yellowish color and round shape mostly found at the posterior pole (Hughes et al. 2007; Virgili et al. 2015). Considering the mechanisms inducing the chronic retinal damage, an alteration of the metabolic sustainment of the photoreceptors cells (rods and cones) and of the retinal pigment epithelium (RPE) is supposed, depending on inflammation processes and vascular modifications (Gehrs et al. 2006; Nowak 2006).

MD is a multi-factorial disease: among the several risk factors involved in its etiology the most important is age (Hyman et al. 2002; Klein et al. 2004). Other recognized are smoke (Clemons et al. 2005; Fujihara et al. 2008), diabetes (Kearney et al. 2014), alcohol abuse (Baird et al. 2014; Chong et al. 2018) and inheritance (Tuo et al. 2004; Yoshimura 2010), while a possible association with female gender (Cho et al. 2014) and other factors such as high C reactive protein levels, low antioxidant vitamins intake, dyslipidemia, fair iris color, previous cataract surgery (Chakravarthy et al. 2010; Ehmann et al. 2017; Gopinath et al. 2015; Kikuchi et al. 2007; Shaw et al. 2016) are supposed. Also an exposure to some chemicals, as lead and iron, was found to be associated with MD (Biesemeier et al. 2015; Hwang et al. 2015; Ugarte et al. 2013). Another MD risk factor is long-term exposure to optical radiation, in particular of the bands in the range of 400–550 nm of wavelength (near ultraviolet—UV-A—and visible “blue-light”), able to induce, in laboratories and animal models, a photochemical damage of the retina due to the formation of oxygen free radicals (OFR) (Sui et al. 2013; Wang et al. 2003). Eye exposure to optical radiation is mainly related to solar radiation and, consequently, outdoor workers (OWs) may be at risk, especially if they work on surfaces able to reflect optical radiation, as water, white sand, snow or shiny metals, as the eye is anatomically quite well protected from the solar rays coming from the sky (ICNIRP 2010; Modenese et al. 2018). Also artificial sources may induce relevant exposures of the eye to optical radiation, as in example indicator lamps and traffic signals, lamps used for the stage lighting and for projections, insect traps, LASERS, welding lights, etc (ICNIRP 2010; Modenese et al. 2016a; Thürauf 1979), but according to the current knowledge the main problems are related to acute eye exposures as a consequence of occupational eye injuries caused by optical radiations, that are quite rare events (Gobba et al. 2017; Kuckelkorn et al. 1995).

Considering these premises, among the various MD risk factors at least optical radiation and chemical exposure may be related to work, but to date the knowledge on specific occupational risk factors and on particular categories of workers at increased risk for MD is scant. It has to be also noted that, currently, the aging of the workforce is a growing problem worldwide (Poscia et al. 2016), and in the next years an increasing number of workers being diagnosed with MD is expected.

For these reasons our aim is to systematically review the recent development of research on the possible work-related risk of MD, studying if particular groups of workers have been found at risk for developing this disease and identifying the specific occupational risk factors detected in the studies and the methods applied to evaluate the exposure.

## Materials and methods

An electronic search in accordance with Preferred Reporting Items for Systematic Reviews and MetaAnalyses (PRISMA) (Liberati et al. 2009) was performed in the Medline (through PubMed) and Scopus databases. Broad limiters were set to include scientific literature covering a period of 50 years, from 1st March 1967 to 1st March 2017. The systematic review was limited to original research articles with an available English abstract published in peer-reviewed journals. Reviews, case reports, comments or letters were not considered. The following search string, modified by previous publications (Mattioli et al. 2010; Modenese et al. 2017), was built: “macular degeneration” AND (worker* OR job* OR occupation*).

Eligible were studies in which an assessment of the job history of patients with a MD diagnosis has been performed, and also studies in which workers have been investigated for the detection of the retinal disease. For the diagnosis of MD we considered clinical diagnosis made by an ophthalmologist and also studies evaluating surgical cases. We did not considered studies in which the subjects self-reported symptoms related to MD without a proved medical diagnosis or in which the cases were not humans.

Considering occupation, we included studies in which the specific occupation of the subjects was evaluated, and also studies considering a generic work categorization, such as “outdoor/indoor worker” or “white collar/blue collar worker”.

Data extraction was performed by one reviewer and checked by another. The extraction was performed by reading all of the available abstracts of the studies returned from the input string in the two databases. Following this, full papers were retrieved for all of the works that met the inclusion criteria. The reference listings of the selected papers were also checked to find other significant research articles.

To assess the quality of the studies, each author independently rated the papers according to a modified version of the Newcastle–Ottawa Scale (Poole et al. 2017). This method results in a score between 0 and 10, that can be assigned to the papers according to the following three domains:

- Selection bias domain: maximum 5 points; Items: (1) Is the sample representative of the population? (2) Was more than one site studied? (3) Was a power calculation undertaken? (4) Did the authors use standardized measurement tools to assess exposures? (5) Did the authors use standardized measurement tools to assess outcomes?
- Comparability domain: maximum 3 points; Items: (1) Were confounding factors assessed? (several considered: two points, some considered: one point, none: zero) (2) Did the study employ an appropriate control group?
- Outcome domain: maximum 2 points; Items: (1) Were statistical tests appropriate? (2) Were conclusions justified?

It was necessarily to adapt the method to our Systematic Review: as we did not have information on power calculation for none of the papers collected, but we could not exclude that this procedure was performed in some of the retrieved studies, we decided not to consider this aspect in the Selection Bias domain and accordingly the maximum total score in our analysis is 9.

In case of disagreement in the evaluation, the two authors reconciled the differences in judgements through discussion.

## Results

### Study selection

**Fig. 1:**
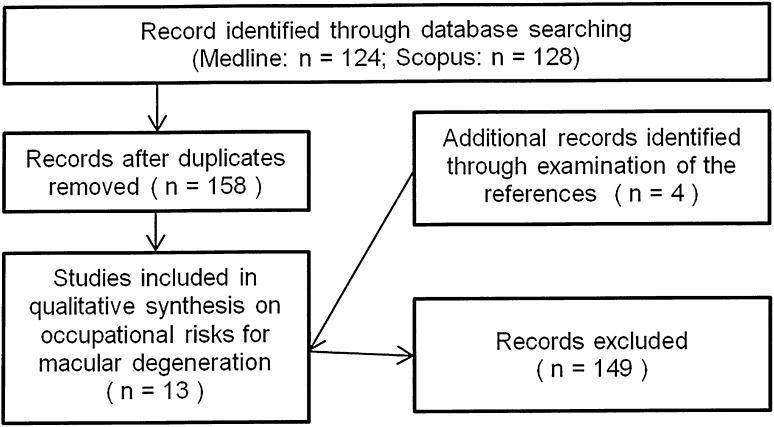
Selection process of the reviewed studies

The literature search resulted in 124 items from Medline and 128 items from Scopus. After the elimination of duplicates 158 articles have been selected. The two authors independently examined the abstracts and agreed on the studies to be included in the review according to criteria described in the “Methods” section 149 papers retrieved were excluded for the following reasons: 67 because they did not investigate, nor estimate, an occupational exposure to a specific risk factor in relation to the outcome of interest (i.e., macular degeneration, MD), 37 and 19 as they were review/letters/comments and because not written in English language, respectively, 19 because did not specifically investigate the outcome of interest (i.e., macular degeneration), 6 because were on animals and, finally 1 as was a case report. 9 on 158 studies were finally selected, and four more studies were identified from a hand search of the references; as a consequence, in this review a total of 13 studies is included (Fig. 1).

### Synthesis of the results

Table 1 summarizes the main characteristics and findings of the 13 studies reviewed, including the occupational risk factors considered and their evaluation methods, and reporting the odds ratio, crude or adjusted for specific confounders. Furthermore, the different types of MD studied, with grading and diagnosing methods applied, when reported by authors, are shown in Table 1, as well as other relevant results of the studies on associations between MD and other factors. Finally, in Table 1 we report also the scoring (range 0–9) attributed to the papers retrieved, based on the quality assessment performed according to an adapted version of the Newcastle–Ottawa Scale (see “Materials and methods” section); the details of the evaluation, including the domains considered, are presented in Table 2.

**Table 1:** Main characteristics and results of the studies included in the systematic review on macular degeneration and exposure to occupational risk factors

First author, year	Place	Subjects group/sample size (*n*)		Occupational risk factor associated (if identified), MD subtype and severity (if provided) and MD frequency (if available), other relevant results of the study	Quality score (0–9)
Retrospective/prospective					
Thapa, 2011	Nepal	PtA/141		SR-OW (based on job title) Sample composition = 42.6% farmers (most frequent job) Dry vs wet MD = 62.4% vs 37.6%; in farmers 55 vs 45% (*p* = 0.077)	2
Njiric, 2007	Croatia	PtA/6617		SR-OW (based on job title) 3-years MD (any grade) incidence = 1.9% OW (total number = 463; 7% of the sample) vs 0.8% IW (*p* < 0.001)	3
Vojnikovic 2007	Croatia	Wo/1371		SR-OW (based on job title) 2-year MD (any grade) incidence = 18% OW (farmers, fishermen = 95% of the sample) vs 2.5% IW 2-year incidence of central vision loss: 21% OW vs 4% IW 2-year incidence of glaucoma (suspected): 28% OW vs 0% IW	2
Klein, 2001	US	GP(A)/3672		5-years incidence of early stage MD = 17% for waiters; 13% for cooks; 21% for bartenders; 13% for cleaning services personnel History of service occupation (based on job title) Waiters, cooks, bartenders, cleaning personnel: OR*= 1.8 (1.01–3.3), compared to white collar workers Blue collar vs white collar workers: OR*= 1.2 (0.9–1.6) Farmers vs white collar: OR*= 0.5 (0.2–1.15) Other results: 13–15 years of education vs < 12: OR*= 0.4 (0.25–0.7) *Adjusted for age and sex	7
Bressler, 1995	US	Wo/483		SR-OW (based on job title): 5-years incidence in Maritime workers = Grade 3 MD*: age adjusted 9% (30–39 ys: 7%; 40–49 ys: 4%; 50–59 ys: 7%; 60–69 ys: 14%; >70 ys: 26%) Grade 4 MD: neovascular disease developed in 1 OW (0.2%), geographic atrophy in none *Classified as eyes with one or more of the following: large or confluent drusen and/or focal hyperpigmentation and/or nongeographic atrophy of the retinal pigmented epithelium Cumulative sunlight exposure of the maritime workers who developed Grade 3 and 4 MD = 0.84 ± 0.63 Maryland sun years (standard annual SR exposure in Maryland, US; based on an integrated method combining subjective data collected with a questionnaire, environmental SR irradiance data with meteorological database and modelisation to determine personal ocular exposure)	8
Cross sectional/case–control					
Saadat, 2012	Iran	PtA/223		SR-OW (based on job title): polymorphism of gene XRCC7 in OW with exudative MD: OR 3.1 (CI 95%, 1.04–9.4; *p* = 0.042), adjusted for age, compares with IW OW = 77 subjects (34.5% of the sample); OW with MD = 51 (23%)	3
Plestina-Borjan, 2007	Croatia	GP(A)/623		SR-OW (based on job title): 113 maritime workers and farmers with SR exposure > 8 h/day had MD, *X*^2^ 186.22, *p* < 0.001 Prevalence of different grades* of MD in 420 OWs. Grade 4: 4.2%; grade 3: 4.2%; grade 2: 14.0%; grade 1: 11% *Grading of MD: same classification as Taylor (1990) (see below)	3
Hyman, 1983	US	PtA/465		Occupational chemical exposure (based on questionnaire investigation), history of: OR* = 4.2 (1.1–15.2). Considering only males: OR* = 3.8 (1.0–14.5) Other associated factors: inheritance OR*= 2.9 (1.5–5.5) Cigarette smoking in males: OR* = 2.6 (1.15–5.75) History of cardiovascular diseases: OR* = 1.9 (1.03–3.34) *Adjusted for age and sex	7
Cross sectional					
Schick, 2016	Europe		PtA/3701	Prevalence = 20.3% early MD*; 31.9% late MD* SR-OW (based on job title and subjective investigation of SR exposure history with a questionnaire): OR** (compared with IW) = 2.6 (1. 9–3.5) for late MD*; n.a. for early MD* Past SR exposure > 8 h/day: OR** (compared with “avoiding the sun”) = 6.3 (1.4–27.5) for early MD; = 2.6 (1.3–5.2) for late MD *Early MD = presence of at least 10 small drusen and pigmentary changes Late MD = either MD with geographic atrophy and/or choroidal neovascularization in at least one eye **Adjusted for age, gender, and smoking behavior	8
Park, 2014	South Korea		GP(A)/14352	Age weighted prevalence: 6.6% for all forms; 6% early MD*; 0.6% all late MD* forms; 0.5% wet late MD*; 0.1% geographic atrophy* Blue collar workers vs white collar OR** = 2.0 (1.5–2.6) Not occupied vs white collar OR** = 1.6 (1.2–2.1) Other risk factors: low education vs high OR**=1.5 (1.2–1.9) Anemia: OR** = 1.4 (1.0–1.9); HBsAg carrier: OR** = 1.9 (1.3–2.7) *Early MD if presence of soft indistinct/reticular drusen, or presence of hard or soft distinct drusen with pigmentary abnormalities. Late MD: wet MD or geographic atrophy (GA). Wet = retinal pigment epithelial detachment or serous detachment of the sensory retina, subretinal or sub-RPE hemorrhages, and subretinal fibrous scars. GA = circular discrete area of retinal depigmentation with visible choroidal vessels **Adjusted for age, gender and smoking status	8
Caljkusic-Mance, 2010	Croatia		PtA/60	Among the MD cases, 75% dry (atrophic) MD, 25% wet (neovascular) MD SR-OW (based on job title): OW (*n* = 42) vs controls: 70 vs 30% (*X*^2^ = 17.6, *p* < 0.0001)	3
Fletcher, 2008	Europe		GP(A)/4753	Prevalence of MD according to severity* in subjects > 65 years: grade 4 MD (neovascular) = 2.3%; grade 4 (geographic atrophy) = 1%; early MD (grade 1 + 2 + 3) = 45.9%; grade 1 = 36.5%; grade 2 = 10.1%; grade 3 = 2.5% SR-OW (based on an integrated method combining subjective data collected with a questionnaire, environmental SR irradiance data with meteorological database and modelisation to determine personal ocular exposure, considering also the blue-light component): subjects with lowest dietary intake of antioxidants and high blue light exposure in central hours of the day OR** = 3.72 (1.56–8.88) for neovascular MD vs atrophic OR** = 1.95 (1.06–3.58) for grade 3 MD vs grade 0 *Grading of MD: same classification as Taylor (1990) (see below) **Adjusted for age, sex, smoking, diabetes, cardiovascular disease, education, aspirin use, retinol, cholesterol	9
Taylor, 1990	US		Wo/782	MD prevalence in maritime workers according to severity = 27.6% grade 1; 18.9%grade 2; 12.0% grade 3; 1.2% grade SR-OW (based on an integrated method combining subjective data collected with a questionnaire, environmental SR irradiance data with meteorological database and modelisation to determine personal ocular exposure, considering also the blue-light component): ocular blue light exposure (past 20 years, OWs > 50 years old) = OR**: 1.35 (1.0–1.8) for grade 4 MD, n. s. considering other MD grades, n. s. considering ocular UV exposure *Classification grade 1 = < 20 small drusen; grade 2 > 20 or more small drusen in central; grade 3 large or confluent drusen, focal hyperpigmentation; grade 4 exudative disease, geographic atrophy **Logistic regression analysis: increase in exposure of 0.1 MSY (standard annual SR exposure in Maryland, US)	8

*GP(A)* general population (adults), *IW* indoor workers, *MD* macular degeneration, *OR* odd ratio (confidence interval 95%), *OW* outdoor workers, *PtA* patients (adults), *n.a*. no association, *n. s*. not significant, *SR* solar radiation exposure, *Wo* workers

**Table 2:** Results of the quality assessment of the studies included in the systematic review on macular degeneration and exposure to occupational risk factors (adapted from Poole et al. 2017)

First author, year	Selection bias domain (max 4 points)				Comparability domain (max 3 points)			Outcome domain (max 2 points)		Total score (0–9)
	Sample representative	> 1 site studied	Standard exposure	Standard outcome	Several confounders	Some confounders	Controls	Statistics appropriate	Conclusions justified	
Retrospective/prospective										
Thapa, 2011	1	0	0	1	0	0	0	0	0	2
Njiric, 2007	1	0	0	1	0	0	1	0	0	3
Vojnikovic, 2007	0	0	0	1	0	0	1	0	0	2
Klein, 2001	1	1	0	1	0	1 age, sex	1	1	1	7
Bressler, 1995	1	1	1	1	0	1 age	1	1	1	8
Cross sectional case–control										
Saadat, 2012	0	0	0	1	0	1 age	1	0	0	3
Plestina-Borjan, 2007	0	1	0	1	0	0	1	0	0	3
Hyman, 1983	1	1	0	1	0	1 age, sex	1	1	1	7
Cross sectional										
Schick, 2016	1	1	0	1	2 age, sex, smoking	0	1	1	1	8
Park, 2014	1	1	0	1	2 age, sex, smoking	0	1	1	1	8
Caljkusic-Mance, 2010	0	1	0	1	0	0	1	0	0	3
Fletcher, 2008	1	1	1	1	2 age, sex, smoke, diabetes, cardio-vascular disease, education, retinol, aspirin, cholesterol	0	1	1	1	9
Taylor, 1990	1	1	1	1	0	1 (age)	1	1	1	8

Of the 13 articles included in our Systematic Review, 10 evaluated the presence of MD in OW exposed to solar radiation (SR). 4 Croatian studies assessed the frequency of MD simply considering occupational SR exposure classifying workers as OW or indoor workers (IW). Njiric et al. (2007) performed a retrospective study including all the patients visiting the Eye Polyclinic of Rijeka in Croatia during the years 1995, 2000 and 2005, for a total of 6617 subjects. The incidence of MD resulted 0.75% in 1995, 0.93% in 2000 and 1.07% in 2005. The patients were divided in two groups according to the outdoor or indoor occupation: the 1.9% of the OW were diagnosed with MD during the 3 years of observation, VS only the 0.8% of the IWs (*p* < 0.001). Vojnikovic et al. (2007) found a higher MD frequency in farmers and fishermen of the Rab island (Adriatic Sea, 44°40′N), within a sample of 1371 subjects aged 45–65 years, followed for a biennium. MD was diagnosed in the 18% of the OW, while only in the 2.5% of the IWs. Plestina-Borjan et al. (2007) conducted a study in 632 subjects over 50 years, of which 420 were mainly fishermen, seamen and farmers from a Croatian island, while the others were from Zagreb city. MD prevalence was higher in OW from the island than in subject from Zagreb, 34.3 vs 16%, respectively, and it was significantly associated with mean daily SR exposure (*X*^2^ = 216.4; *p* = 0.000). Caljkusic-Mance et al. (2010) evaluated the occupation history in a sample of 60 patients, median age was 70.2 (range 52–86), diagnosed with dry or wet MD during years 2008 and 2009 in an ophthalmologic clinic in Croatia. 42 cases were OW (70%) and 18 patients were IW (30%) (*p* < 0.0001).

Another hospital-based 9 months prospective study from Nepal (Thapa et al. 2011) considered the occupation of a sample of patients representing all the consecutive cases of MD diagnosed from September 2008 to May 2009 at the local institute of ophthalmology. A total of 141 patients were recruited (mean age 69.5 years) and, considering work activity, the 42.5% of the sample were agriculture workers (*p* = 0.077) with occupational SR exposure.

In the previous studies workers were simply classified as outdoor or indoor workers, while in the following studies a detailed assessment of SR exposure has been performed. In a recent multi-centric European study conducted by Shick et al. (2016), SR exposure and job history were investigated with a detailed questionnaire. The results showed a prevalence of MD in general population of 20.3% for early MD and 31.9% for late MD. MD was not found to be associated with current SR exposure, but both, early and late MD, proved association with a history of past sunlight exposure major than 8 h outdoor per day, typical of outdoor work, with an OR for early MD of 5.54 (95% CI 1.25–24.58), and of 2.77 (95% CI 1.25–6.16, *p* = 0.01) for late MD. Furthermore, OW was more likely to have late MD with an OR of 2.57 (1.89–3.48), after adjustment for age, gender, and smoking behavior, while no association with early MD was found. In another multi-centric European study (Fletcher et al. 2008), a more detailed method for SR exposure evaluation was adopted. In 4753 participants, aged 65 years or older, fundus photography was collected and in 101 individuals neovascular MD was diagnosed, in 2182 early MD was found and 2117 subjects were classified as controls. All subjects were interviewed for adult lifetime sunlight exposure, and gave blood for antioxidant analysis. SR exposure was estimated by combining meteorological and questionnaire data. The questionnaire evaluated the history of sunlight exposure in various occupational periods of life, investigating for each period the number of hours spent outdoor between 9 am and 5 pm, and specifically between 11 am and 3 pm, and the adoption of protective equipment such as hat and sunglasses. For all residences of 1 year or longer, ambient UVB and UVA were estimated from environmental databases, and blue light was estimated using a radiation model that estimates spectral radiation as a function of time of day, day of the year, and latitude; exposure was adjusted for coefficients for cloud cover, surfaces, and protections. The Authors did not report a direct association between SR exposure in outdoor workers and MD, but observed a significant association in subjects with the lowest dietary intake of antioxidants and high blue light exposure in midday hours with an OR of 1.95 (1.06–3.58) for grade 3 MD vs grade 0.

History of cumulative exposure to the blue light component of SR exposure was found to be associated with severe MS (grade 4) also in the “watermen study” performed in Maryland, US, by Taylor et al. (1990) in late 80 s. 838 maritime workers underwent an ophthalmologic examination and grade 4 MD showed a prevalence of 1.2%. Cumulative sunlight exposure was evaluated with a mixed model, including laboratory measurements of eye exposure, environmental data available through meteorological databases and a questionnaire administration. This method estimated the exposure for the different optical radiation bands of SR, UVA, UVB and blue light and the data was reported as a fraction of a standard “Maryland Sun-Year”, representing the mean SR exposure in 1 year typical of this US country: grade 4 MD was significantly more frequent in watermen with an increasing of 0.1 “Maryland Sun Years” of blue light exposure for a period of 20 years, OR 1.35 (1.0–1.81), while no significant association was found for the UV components. This study represented the baseline evaluation for the longitudinal study of Bressler et al. (1995), aimed to evaluate the 5-year MD incidence in 483 Maritime workers who underwent a follow-up examination. The MD incidence increased with OWs age: 7% in the age group 50–59 years (ys), 14% in 60–69 ys and 26% in > 70 ys. Also in this study SR exposure was evaluated with the same semi-quantitative method used by Taylor et al., and cumulative SR exposure resulted 0.84 ± 0.63 “Maryland sun years” in the group of maritime workers followed.

Finally, a different type of study investigating a sample of 111 patients with exudative MD classified as outdoor or indoor workers according to their job titles was performed in Iran by Saadat et al. (2012). The aim of the study was to investigate the presence of polymorphism of the Gene XRCC7, located on human chromosome 8q12, where contiguous markers possibly associated with MD have previously been identified. This gene encodes the catalytic subunit of a nuclear DNA-dependent serine/threonine protein kinase, contributing in the recognition and repair of DNA double-strand breaks, found to be associated with cancer by other Authors. Saadat et al. found that the presence of gene XRCC7 polymorphism was significantly highly expressed in OWs than in indoor workers with MD, OR 3.1 (1.04–9.39), *p* = 0.042.

Moving now to possible other occupational MD risk factors, two studies investigated the occupation activity in large samples of population, but without hypothesizing a specific factor involved. Klein et al. (2001) performed a longitudinal examination of the cohort of the “Beaver Dam Eye Study”, Wisconsin—US—, composed by 3681 adults (range 43–86 years of age at baseline). Status and type of employment were investigated with a questionnaire and fundus photography was collected to diagnose MD. Blue collar workers compared with white collars were more luckily to have early MD (*p* < 0.05), and in particular a higher 5-year MD incidence was observed in waiters (17%), cooks (13%) and bartenders (21%), and in personnel involved in cleaning services (13%) versus other working categories, including farmers (OR 1.83, CI 95% 1.01–3.32). Also in the South-Korean study of Park et al. (2014) the prevalence of MD was found to be higher in blue collar workers than in white collars, with a significant OR of 1.82 (95% CI 1.37–2.42, *p* < 0.001). This study was conducted from 2008 to 2011 in 14,352 participants over 40 years of age examined with fundus photographs, diagnosing MD in the 6.6% of the sample, of which 6% early MD and 0.6% late MD. Demographic and socioeconomic factors were investigated with a questionnaire; no association with sun exposure evaluated independently of the job, as major than 5 h per day pent in the sun, was found.

Finally the case-control study performed in Baltimore, U.S., by Hyman et al. (1983) considered 162 cases of MD and 175 controls matched by age and sex. Study participants were examined with fundus photographs and interviewed for past medical, residential, occupational, smoking and family histories, as well as social and demographic factors. Diagnoses were validated by means of fundus photographs. A statistically significant association was shown between MD and non-specific occupational chemical exposure investigated with the question “Did you ever work around chemicals which caused your eyes to burn, on a regular basis?”, OR 4.2 (95% CI 1.1–15.2).

## Discussion

### Main findings of the studies reviewed

In most studies included in this systematic review (10/13) the risk related to occupational solar radiation exposure was evaluated; all the ten studies found a positive association between SR exposure and MD. These results are in agreement with scientific literature, showing an OR of 2.09 (95% CI 1.19–3.65) for SR exposure and early MD (Cruickshanks et al. 2001), and a relative risk of 2.20 (95% CI 1.02–4.73) (Tomany et al. 2005) in general population; these studies have been also included in a recent systematic review and meta-analysis (Sui et al. 2013) of 14 studies, 12 of which showed an increased risk of MD for high levels of SR exposure, and in 6 cases the associations were statistically significant. In six studies the occupational SR exposure was evaluated simply classifying workers as outdoor or indoor, while in four studies the exposure was evaluated with a more detailed method, considering subjective and objective data: two of these studies estimated, among the SR components, the contribution of different optical bands and found a specific association between MD and cumulative blue light exposure, not for the UV components. It has to be considered that, among the ultraviolet component, only a part of UV-A from 380 to 400 nanometers (nm) of wavelength can reach the retina with a possible chronic photochemical damage, especially in younger ages, while UV bands below 380 nm are absorbed in the anterior eye (Sliney 2002). The other optical bands of SR able to interact with macular cells with photochemical mechanisms are the visible “blue-light” bands between 400 and 550 nm of wavelength. The remaining part of the visible spectrum, as well as the infrared bands, reach the retina, but the interactions mechanisms are based on the possible thermal effects, mainly relevant in inducing acute disorders (ICNIRP 2004; Sliney 2002; Sliney et al. 2005).

Quite surprisingly, we found no studies evaluating the presence of MD in groups of workers exposed to artificial optical radiation, such as welders (Maier et al. 2005; Tenkate 2017), health personnel (Price et al. 2016) and others. It has to be noted that, as in example, in Europe, according to the Directive 2006/25/EC, for artificial optical radiation exposure specific occupational limits for blue light and UV have to be respected to protect the eye (ICNIRP 2004; Sliney 2002; Sliney et al. 2005), as well as for the skin (ICNIRP 2004; Modenese et al. 2016b; Ulrich et al. 2016).

The other work-related risk for MD found in this review is a non-specific history of chemical exposure at work, found to be associated with MD in a 1983 U.S. study (Hyman et al. 1983). This result may be supported by some more recent studies suggesting that chemicals like lead and iron can accumulate in the macula inducing a chronic damage, while the depletion of fundamental chemicals like zinc may play a role in the failure of the protective antioxidant mechanisms (Biesemeier et al. 2015; Hwang et al. 2015; Ugarte et al. 2013).

Other two studies reviewed did not evaluate specific occupational risk factors, finding an association between MD and the general category of “blue-collar” workers: SR exposure may be involved, but also chemical exposure and possibly other factors; in the longitudinal U.S. study by Klein et al. the specific “blue collar” categories at higher risk were that of waiters, cooks, bartenders and cleaning personnel, and it is not clear what occupational risk factor can be involved, even if, at least for cleaning personnel, chemical exposure may be considered.

### Limitations of the review

The quality of the analysis performed in the studies reviewed is rather inhomogeneous, and some weak aspects can be observed. On the other hand, especially considering the scarce number of studies published, to analyze all the literature of interest, we decided not to exclude any pertinent study on the topic of “occupational risk factors for macular degeneration”. Another problem is that the study designs applied by researchers are quite different and scarcely comparable, precluding any possibility of a meta-analysis. Based on our quality assessment, 6 on 13 studies showed a rather poor quality, presenting problems in particular in the exposure assessment phase and in the selection of the sample. These six studies (Caljkusic-Mance et al. 2010; Njiric et al. 2007; Plestina-Borjan et al. 2007; Saadat et al. 2012; Thapa et al. 2011; Vojnikovic et al. 2007) considered MD and SR exposure of outdoor workers. The main issue in all these studies is the exposure evaluation that was only based on job title; moreover, in some studies is not clear the criteria for enrollment and for assignation of the subjects to the OW or IW groups. Also the statistical analysis performed in these six studies is not adequate to fully support their conclusions. On the other hand, four very well designed studies, including a large number of subjects and a solid methodology for the assessment of both outcome and exposure, support an association between MD and occupational sunlight exposure, especially considering the cumulative ocular blue-light exposure (Bressler et al. 1995; Fletcher et al. 2008; Schick et al. 2016; Taylor et al. 1990). Other three well designed studies, with representative samples, good outcome definition and adequate statistics (Hyman et al. 1983; Klein et al. 2001; Park et al. 2014) consider other possible occupational risk factors, but the exposure assessment is based on a subjective evaluation, not allowing any affordable inference regarding a possible association between the investigated occupational risk factors (e.g., chemical exposure) and MD. This topic certainly deserves further research with more adequate methods.

## Conclusions

To date few studies have examined occupational risk factors associated with MD, as well as few studies evaluated the frequency of this disease in specific working groups. Nevertheless, available data support the hypothesis of an association between long-term occupational SR exposure, in particular for its blue-light component, and MD in outdoor workers. According to the high number of OWs worldwide (e.g., about 15 million only in Europe) and to the high prevalence of the disease in people aged 50 years or more, these results suggest the opportunity of specific organizational and individual protective measures to prevent this disease, possibly including a medical examinations of the workers’ eyes. No studies on the relations between occupational exposures to artificial light and MD were found, while some studies suggest that occupational exposure to chemicals may represent a possible risk factor for MD. Overall, the scarce number of studies, and their inhomogeneous quality, supports the need of further research on the possible association between MD and occupational risk factors.

## Electronic supplementary material

Below is the link to the electronic supplementary material.

Supplementary material 1 (DOCX 46.906 kb)
