# Supplementary material for: Macular degeneration and occupational risk factors: a systematic review
Source: Int Arch Occup Environ Health. 2018 Sep 6;92(1):1–11. doi: 10.1007/s00420-018-1355-y (PMC6323067; doi:10.1007/s00420-018-1355-y)
Supplement: Supplementary file 1 — Supplementary material 1 (DOCX 46.906 kb) [file 420_2018_1355_MOESM1_ESM.docx]

**FULL LIST OF PAPERS RETRIEVED FROM PUBMED AND SCOPUS (AFTER DUPLICATED REMOVED) FOR THE QUERY "macular degeneration” AND (worker* OR job* OR occupation*) WITH REASONS OF EXCLUSIONS**

**- Scientific Literature Analyzed from 1st March 1967 to 1st March 2017**

**PUBMED RESULTS**

1: Copolillo A, Christopher A, Lyons A. Effects of Simulated Low Vision on

Postural Adjustment to Changes in Center of Mass in Older Adults. Occup Ther

Health Care. 2017 Feb 3:1-11. doi: 10.1080/07380577.2016.1278295. [Epub ahead of

print] PubMed PMID: 28156182. **EXCLUDED, NOT SPECIFICALLY INVESTIGATING OUR OUTCOME**

2: Gorusupudi A, Nelson K, Bernstein PS. The Age-Related Eye Disease 2 Study:

Micronutrients in the Treatment of Macular Degeneration. Adv Nutr. 2017 Jan

17;8(1):40-53. doi: 10.3945/an.116.013177. Review. PubMed PMID: 28096126; PubMed

Central PMCID: PMC5227975. **EXCLUDED, REVIEW-LETTER-COMMENT**

3: Pozet A, Lejeune C, Bonnet M, Dabakuyo S, Dion M, Fagnoni P, Gaimard M, Imbert

G, Nerich V, Foubert A, Chotard M, Bonin M, Anota A, Bonnetain F. Evaluation of

efficacy and efficiency of a pragmatic intervention by a social worker to support

informal caregivers of elderly patients (The ICE Study): study protocol for a

randomized controlled trial. Trials. 2016 Nov 3;17(1):531. PubMed PMID: 27881145;

PubMed Central PMCID: PMC5122007. **EXCLUDED, NOT SPECIFICALLY INVESTIGATING OUR OUTCOME**

4: Zetterlund C, Richter HO, Lundqvist LO. Visual, Musculoskeletal, and Balance

Complaints in AMD: A Follow-Up Study. J Ophthalmol. 2016;2016:2707102. PubMed

PMID: 27830084; PubMed Central PMCID: PMC5088334. **EXCLUDED, NOT INVESTIGATING/ESTIMATING OCCUPATIONAL EXPOSURE**

5: Krigel A, Berdugo M, Picard E, Levy-Boukris R, Jaadane I, Jonet L,

Dernigoghossian M, Andrieu-Soler C, Torriglia A, Behar-Cohen F. Light-induced

retinal damage using different light sources, protocols and rat strains reveals

LED phototoxicity. Neuroscience. 2016 Dec 17;339:296-307. doi:

10.1016/j.neuroscience.2016.10.015. PubMed PMID: 27751961. **EXCLUDED, STUDY IN ANIMALS**

6: Shen XF, Huang P, Fox DA, Lin Y, Zhao ZH, Wang W, Wang JY, Liu XQ, Chen JY,

Luo WJ. Adult lead exposure increases blood-retinal permeability: A risk factor

for retinal vascular disease. Neurotoxicology. 2016 Dec;57:145-152. doi:

10.1016/j.neuro.2016.09.013. PubMed PMID: 27663850. **EXCLUDED, NOT SPECIFICALLY INVESTIGATING OUR OUTCOME**

7: Chong MF, Jackson AJ, Wolffsohn JS, Bentley SA. An update on the

characteristics of patients attending the Kooyong Low Vision Clinic. Clin Exp

Optom. 2016 Nov;99(6):555-558. doi: 10.1111/cxo.12395. PubMed PMID: 27320822**. EXCLUDED, NOT INVESTIGATING/ESTIMATING OCCUPATIONAL EXPOSURE**

8: Bowen M, Edgar DF, Hancock B, Haque S, Shah R, Buchanan S, Iliffe S, Maskell

S, Pickett J, Taylor JP, O’Leary N. The Prevalence of Visual Impairment in People

with Dementia (the PrOVIDe study): a cross-sectional study of people aged 60–89

years with dementia and qualitative exploration of individual, carer and

professional perspectives. Southampton (UK): NIHR Journals Library; 2016 Jul.

PubMed PMID: 27489923. **EXCLUDED, NOT INVESTIGATING/ESTIMATING OCCUPATIONAL EXPOSURE**

9: Wu W, Weng Y, Guo X, Feng L, Xia H, Jiang Z, Lou J. The Association Between

Serum Vitamin D Levels and Age-Related Macular Degeneration: A Systematic

Meta-Analytic Review. Invest Ophthalmol Vis Sci. 2016 Apr 1;57(4):2168-77. doi:

10.1167/iovs.15-18218. PubMed PMID: 27111565. **EXCLUDED, REVIEW-LETTER-COMMENT**

10: Warren M, DeCarlo DK, Dreer LE. Health Literacy in Older Adults With and

Without Low Vision. Am J Occup Ther. 2016 May-Jun;70(3):7003270010p1-7. doi:

10.5014/ajot.2016.017400. PubMed PMID: 27089294. **EXCLUDED, NOT INVESTIGATING/ESTIMATING OCCUPATIONAL EXPOSURE**

11: Price RB, Labrie D, Bruzell EM, Sliney DH, Strassler HE. The dental curing

light: A potential health risk. J Occup Environ Hyg. 2016 Aug;13(8):639-46. doi:

10.1080/15459624.2016.1165822. PubMed PMID: 27003737. **EXCLUDED, NOT SPECIFICALLY INVESTIGATING OUR OUTCOME**

12: Shim SH, Kim SG, Bae JH, Yu HG, Song SJ. Risk Factors for Progression of

Early Age-Related Macular Degeneration in Koreans. Ophthalmic Epidemiol.

2016;23(2):80-7. doi: 10.3109/09286586.2015.1129425. PubMed PMID: 26950426.

**EXCLUDED, NOT INVESTIGATING/ESTIMATING OCCUPATIONAL EXPOSURE**

13: Thapa R, Bajimaya S, Paudyal G, Khanal S, Tan S, Thapa SS, van Rens G.

Population awareness of diabetic eye disease and age related macular degeneration

in Nepal: the Bhaktapur Retina Study. BMC Ophthalmol. 2015 Dec 29;15:188. doi:

10.1186/s12886-015-0175-z. PubMed PMID: 26714483; PubMed Central PMCID:

PMC4696239. **EXCLUDED, NOT INVESTIGATING/ESTIMATING OCCUPATIONAL EXPOSURE**

14: Chorley AC, Evans BJ, Benwell MJ. Solar Eye Protection Practices of Civilian

Aircrew. Aerosp Med Hum Perform. 2015 Nov;86(11):953-61. doi:

10.3357/AMHP.4357.2015. PubMed PMID: 26564760. **EXCLUDED, NOT SPECIFICALLY INVESTIGATING OUR OUTCOME**

15: Schick T, Ersoy L, Lechanteur YT, Saksens NT, Hoyng CB, den Hollander AI,

Kirchhof B, Fauser S. HISTORY OF SUNLIGHT EXPOSURE IS A RISK FACTOR FOR

AGE-RELATED MACULAR DEGENERATION. Retina. 2016 Apr;36(4):787-90. doi:

10.1097/IAE.0000000000000756. PubMed PMID: 26441265. **INCLUDED**

16: Ord LM, Wright J, DeAngelis MM, Feehan M. Quality of Life with Macular

Degeneration Is Not as Dark as It May Seem: Patients' Perceptions of the MacDQoL

Questionnaire. J Clin Med. 2015 Sep 22;4(9):1841-52. doi: 10.3390/jcm4091841.

PubMed PMID: 26402711; PubMed Central PMCID: PMC4600162. **EXCLUDED, NOT INVESTIGATING/ESTIMATING OCCUPATIONAL EXPOSURE**

17: Qiu M, Shields CL. Choroidal Nevus in the United States Adult Population:

Racial Disparities and Associated Factors in the National Health and Nutrition

Examination Survey. Ophthalmology. 2015 Oct;122(10):2071-83. doi:

10.1016/j.ophtha.2015.06.008. PubMed PMID: 26255109. **EXCLUDED, NOT SPECIFICALLY INVESTIGATING OUR OUTCOME**

18: Kraljević Pavelić S, Klobučar M, Sedić M, Micek V, Gehrig P, Grossman J,

Pavelić K, Vojniković B. UV-induced retinal proteome changes in the rat model of

age-related macular degeneration. Biochim Biophys Acta. 2015 Sep;1852(9):1833-45.

doi: 10.1016/j.bbadis.2015.06.006. PubMed PMID: 26071645. **EXCLUDED, STUDY IN ANIMALS**

19: Gao G, Ouyang C, Dai J, Xue F, Wang X, Zou L, Chen M, Ma F, Yu M. Baseline

traits of patients presenting at a low vision clinic in Shanghai, China. BMC

Ophthalmol. 2015 Mar 3;15:16. doi: 10.1186/s12886-015-0013-3. PubMed PMID:

25884841; PubMed Central PMCID: PMC4357209. **EXCLUDED, NOT INVESTIGATING/ESTIMATING OCCUPATIONAL EXPOSURE**

20: Vessey KA, Waugh M, Jobling AI, Phipps JA, Ho T, Trogrlic L, Greferath U,

Fletcher EL. Assessment of retinal function and morphology in aging Ccl2 knockout

mice. Invest Ophthalmol Vis Sci. 2015 Jan 27;56(2):1238-52. doi:

10.1167/iovs.14-15334. PubMed PMID: 25626968. **EXCLUDED, STUDY IN ANIMALS**

21: Iejima D, Nakayama M, Iwata T. HTRA1 Overexpression Induces the Exudative

Form of Age-related Macular Degeneration. J Stem Cells. 2015;10(3):193-203.

PubMed PMID: 27125063. **EXCLUDED, NOT INVESTIGATING/ESTIMATING OCCUPATIONAL EXPOSURE**

22: He J, Lu L, Zou H, He X, Li Q, Wang W, Zhu J. Prevalence and causes of visual

impairment and rate of wearing spectacles in schools for children of migrant

workers in Shanghai, China. BMC Public Health. 2014 Dec 22;14:1312. doi:

10.1186/1471-2458-14-1312. PubMed PMID: 25535407; PubMed Central PMCID:

PMC4364498. **EXCLUDED, NOT INVESTIGATING/ESTIMATING OCCUPATIONAL EXPOSURE**

23: Jobling AI, Guymer RH, Vessey KA, Greferath U, Mills SA, Brassington KH, Luu

CD, Aung KZ, Trogrlic L, Plunkett M, Fletcher EL. Nanosecond laser therapy

reverses pathologic and molecular changes in age-related macular degeneration

without retinal damage. FASEB J. 2015 Feb;29(2):696-710. doi:

10.1096/fj.14-262444. PubMed PMID: 25392267. **EXCLUDED, NOT INVESTIGATING/ESTIMATING OCCUPATIONAL EXPOSURE**

24: Schmid MK, Bachmann LM, Fäs L, Kessels AG, Job OM, Thiel MA. Efficacy and

adverse events of aflibercept, ranibizumab and bevacizumab in age-related macular

degeneration: a trade-off analysis. Br J Ophthalmol. 2015 Feb;99(2):141-6. doi:

10.1136/bjophthalmol-2014-305149. Review. PubMed PMID: 25271911. **EXCLUDED, REVIEW-LETTER-COMMENT**

25: Randolph SA. Age-related macular degeneration. Workplace Health Saf. 2014

Aug;62(8):352. doi: 10.3928/21650799-20140708-06. PubMed PMID: 25093372.

**EXCLUDED, REVIEW-LETTER-COMMENT**

26: Fletcher EL, Jobling AI, Greferath U, Mills SA, Waugh M, Ho T, de Iongh RU,

Phipps JA, Vessey KA. Studying age-related macular degeneration using animal

models. Optom Vis Sci. 2014 Aug;91(8):878-86. doi: 10.1097/OPX.0000000000000322.

Review. PubMed PMID: 24978866; PubMed Central PMCID: PMC4186726. **EXCLUDED, REVIEW-LETTER-COMMENT**

27: Barleon L, Wahl J, Morfeld P, Deters C, Lichtmeβ A, Haas-Brähler S, Müller U,

Breitstadt R, Pfeiffer N. The Evonik-Mainz-Eye-Care-Study (EMECS): design and

execution of the screening investigation. PLoS One. 2014 Jun 10;9(6):e98538. doi:

10.1371/journal.pone.0098538. Erratum in: PLoS One. 2014;9(8):e107083. PubMed

PMID: 24915063; PubMed Central PMCID: PMC4051641. **EXCLUDED, NOT INVESTIGATING/ESTIMATING OCCUPATIONAL EXPOSURE**

28: Park SJ, Lee JH, Woo SJ, Ahn J, Shin JP, Song SJ, Kang SW, Park KH;

Epidemiologic Survey Committee of the Korean Ophthalmologic Society.. Age-related

macular degeneration: prevalence and risk factors from Korean National Health and

Nutrition Examination Survey, 2008 through 2011. Ophthalmology. 2014

Sep;121(9):1756-65. doi: 10.1016/j.ophtha.2014.03.022. PubMed PMID: 24813632.

**INCLUDED**

29: Vessey KA, Greferath U, Aplin FP, Jobling AI, Phipps JA, Ho T, De Iongh RU,

Fletcher EL. Adenosine triphosphate-induced photoreceptor death and retinal

remodeling in rats. J Comp Neurol. 2014 Sep 1;522(13):2928-50. doi:

10.1002/cne.23558. PubMed PMID: 24639102; PubMed Central PMCID: PMC4265795. **EXCLUDED, STUDY IN ANIMALS**

30: Shibuya M. VEGF-VEGFR Signals in Health and Disease. Biomol Ther (Seoul).

2014 Jan;22(1):1-9. doi: 10.4062/biomolther.2013.113. Review. PubMed PMID:

24596615; PubMed Central PMCID: PMC3936422. **EXCLUDED, REVIEW-LETTER-COMMENT**

31: Hong SP, Park H, Kwon JS, Yoo E. Effectiveness of eccentric viewing training

for daily visual activities for individuals with age-related macular

degeneration: a systematic review and meta-analysis. NeuroRehabilitation.

2014;34(3):587-95. doi: 10.3233/NRE-141055. Review. PubMed PMID: 24463233. **EXCLUDED, REVIEW-LETTER-COMMENT**

32: Ferronato L, Ukovic A. Enabling positive work outcomes for people with low

vision: two case studies. Work. 2014 Jan 1;47(3):381-6. doi: 10.3233/WOR-131770.

PubMed PMID: 24284659. **EXCLUDED, NOT INVESTIGATING/ESTIMATING OCCUPATIONAL EXPOSURE**

33: Gicquel MC, Tanguy M, Apicella C, Charvier M, Clavaud H, Lescarret B, Neme B,

Perrot P. [The treatment of age-related macular degeneration (AMD) in practice].

Sante Publique. 2013 May-Jun;25(3):315-24. French. PubMed PMID: 24007907. **EXLUDED, ARTICLE NOT IN ENGLISH LANGUAGE**

34: Makou E, Herbert AP, Barlow PN. Functional anatomy of complement factor H.

Biochemistry. 2013 Jun 11;52(23):3949-62. doi: 10.1021/bi4003452. Review. PubMed

PMID: 23701234. **EXCLUDED, REVIEW-LETTER-COMMENT**

35: Thapa R, Poudyal G, Crandall AS, Tabin G. Vitreo-retinal disorders at high

altitude in Nepal. Nepal J Ophthalmol. 2013 Jan-Jun;5(1):57-62. doi:

http://dx.doi.org/10.3126/nepjoph.v5i1.7823. PubMed PMID: 23584648. **EXCLUDED, NOT INVESTIGATING/ESTIMATING OCCUPATIONAL EXPOSURE**

36: Taino G, Paraluppi P, Giorgi M, D'Orso MI, Piccoli B. [Occupational diseases

caused by artificial optical radiation (AOR)]. Med Lav. 2013 Jan-Feb;104(1):3-23.

Review. Italian. PubMed PMID: 23520883. **EXLUDED, ARTICLE NOT IN ENGLISH LANGUAGE**

37: Saadat I, Vakili-Ghartavol R, Farvardin-Jahromi M, Saadat M. Association

between exudative age-related macular degeneration and the G6721T polymorphism of

XRCC7 in outdoor subjects. Korean J Ophthalmol. 2012 Dec;26(6):423-7. doi:

10.3341/kjo.2012.26.6.423. PubMed PMID: 23204796; PubMed Central PMCID:

PMC3506815. **INCLUDED**

38: Vessey KA, Greferath U, Jobling AI, Phipps JA, Ho T, Waugh M, Fletcher EL.

Ccl2/Cx3cr1 knockout mice have inner retinal dysfunction but are not an

accelerated model of AMD. Invest Ophthalmol Vis Sci. 2012 Nov 27;53(12):7833-46.

doi: 10.1167/iovs.12-10650. PubMed PMID: 23074204. **EXCLUDED, STUDY IN ANIMALS**

39: O'Brien EE, Greferath U, Vessey KA, Jobling AI, Fletcher EL. Electronic

restoration of vision in those with photoreceptor degenerations. Clin Exp Optom.

2012 Sep;95(5):473-83. doi: 10.1111/j.1444-0938.2012.00783.x. Review. PubMed

PMID: 22823954. **EXCLUDED, REVIEW-LETTER-COMMENT**

40: Dawson WJ. Bassoonists' medical problems-current state of knowledge. Med

Probl Perform Art. 2012 Jun;27(2):107-12. Review. PubMed PMID: 22739824. **EXCLUDED, REVIEW-LETTER-COMMENT**

41: Thapa R, Paudyal G, Shrestha MK, Gurung R, Ruit S. Age-related macular

degeneration in Nepal. Kathmandu Univ Med J (KUMJ). 2011 Jul-Sep;9(35):165-9.

PubMed PMID: 22609500. **INCLUDED**

42: Arbesman M, Mosley LJ. Systematic review of occupation- and activity-based

health management and maintenance interventions for community-dwelling older

adults. Am J Occup Ther. 2012 May-Jun;66(3):277-83. doi:

10.5014/ajot.2012.003327. Review. PubMed PMID: 22549592. **EXCLUDED, REVIEW-LETTER-COMMENT**

43: Whitson HE, Steinhauser K, Ammarell N, Whitaker D, Cousins SW, Ansah D,

Sanders LL, Cohen HJ. Categorizing the effect of comorbidity: a qualitative study

of individuals' experiences in a low-vision rehabilitation program. J Am Geriatr

Soc. 2011 Oct;59(10):1802-9. doi: 10.1111/j.1532-5415.2011.03602.x. PubMed PMID:

22091493; PubMed Central PMCID: PMC3662468. **EXCLUDED, NOT INVESTIGATING/ESTIMATING OCCUPATIONAL EXPOSURE**

44: Nwosu SN. Age-related macular degeneration in Onitsha, Nigeria. Niger J Clin

Pract. 2011 Jul-Sep;14(3):327-31. doi: 10.4103/1119-3077.86778. PubMed PMID:

22037079. **EXCLUDED, NOT INVESTIGATING/ESTIMATING OCCUPATIONAL EXPOSURE**

45: Markowitz M, Markowitz RE, Markowitz SN. The multi-disciplinary nature of low

vision rehabilitation--a case report. Work. 2011;39(1):63-6. doi:

10.3233/WOR-2011-1151. PubMed PMID: 21673429. **EXCLUDED, NOT INVESTIGATING/ESTIMATING OCCUPATIONAL EXPOSURE**

46: Walker DP, Vollmer-Snarr HR, Eberting CL. Ocular hazards of blue-light

therapy in dermatology. J Am Acad Dermatol. 2012 Jan;66(1):130-5. doi:

10.1016/j.jaad.2010.11.040. PubMed PMID: 21536341. **EXCLUDED, NOT INVESTIGATING/ESTIMATING OCCUPATIONAL EXPOSURE**

47: Fletcher EL, Jobling AI, Vessey KA, Luu C, Guymer RH, Baird PN. Animal models

of retinal disease. Prog Mol Biol Transl Sci. 2011;100:211-86. doi:

10.1016/B978-0-12-384878-9.00006-6. Review. PubMed PMID: 21377628. **EXCLUDED, REVIEW-COMMENT-LETTER**

48: Caljkusić-Mance T, Kovacević D, Novak-Stroligo M, Alpeza-Dunato Z.

Distribution of age-related macular degeneration in Primorsko-Goranska County.

Coll Antropol. 2010 Apr;34 Suppl 2:109-11. PubMed PMID: 21302709. **INCLUDED**

49: Vojniković B, Vojniković D. Benedictine monastery on the Island of Rab hides

the mystery of sunlight influence on development of macular degeneration (AMD).

Is a harmful sunlight, risk factor or cause of macular degeneration? Coll

Antropol. 2010 Apr;34 Suppl 2:5-8. PubMed PMID: 21302698. **EXCLUDED, NOT INVESTIGATING/ESTIMATING OCCUPATIONAL EXPOSURE**

50: Miyamoto N, Izumi H, Miyamoto R, Bin H, Kondo H, Tawara A, Sasaguri Y, Kohno

K. Transcriptional regulation of activating transcription factor 4 under

oxidative stress in retinal pigment epithelial ARPE-19/HPV-16 cells. Invest

Ophthalmol Vis Sci. 2011 Mar 2;52(3):1226-34. doi: 10.1167/iovs.10-5775. PubMed

PMID: 21087962. **EXCLUDED, NOT INVESTIGATING/ESTIMATING OCCUPATIONAL EXPOSURE**

51: Richard AI. Causes of blindness and low vision in Bayelsa State, Nigeria: a

clinic based study. Nig Q J Hosp Med. 2010 Jul-Sep;20(3):125-8. PubMed PMID:

21033320. **EXCLUDED, NOT INVESTIGATING/ESTIMATING OCCUPATIONAL EXPOSURE**

52: Christoforidis JB, Tecce N, Dell'Omo R, Mastropasqua R, Verolino M,

Costagliola C. Age related macular degeneration and visual disability. Curr Drug

Targets. 2011 Feb;12(2):221-33. PubMed PMID: 20887239. **EXCLUDED, NOT INVESTIGATING/ESTIMATING OCCUPATIONAL EXPOSURE**

53: Bekibele CO, Ajav R, Asuzu MC. Eye health of professional drivers of a

Nigerian University. Niger Postgrad Med J. 2009 Dec;16(4):256-9. PubMed PMID:

20037620. **EXCLUDED, NOT INVESTIGATING/ESTIMATING OCCUPATIONAL EXPOSURE**

54: Friberg TR, Brennen PM, Freeman WR, Musch DC; PTAMD Study Group..

Prophylactic treatment of age-related macular degeneration report number 2:

810-nanometer laser to eyes with drusen: bilaterally eligible patients.

Ophthalmic Surg Lasers Imaging. 2009 Nov-Dec;40(6):530-8. doi:

10.3928/15428877-20091030-01. PubMed PMID: 19928717. **EXCLUDED, NOT INVESTIGATING/ESTIMATING OCCUPATIONAL EXPOSURE**

55: Topouzis F, Anastasopoulos E, Augood C, Bentham GC, Chakravarthy U, de Jong

PT, Rahu M, Seland J, Soubrane G, Tomazzoli L, Vingerling JR, Vioque J, Young IS,

Fletcher AE. Association of diabetes with age-related macular degeneration in the

EUREYE study. Br J Ophthalmol. 2009 Aug;93(8):1037-41. doi:

10.1136/bjo.2008.146316. PubMed PMID: 19429584. **EXCLUDED, NOT INVESTIGATING/ESTIMATING OCCUPATIONAL EXPOSURE**

56: Kammer R, Sell C, Jamara RJ, Kollbaum E. Survey of optometric low vision

rehabilitation training methods for the moderately visually impaired. Optometry.

2009 Apr;80(4):185-92. doi: 10.1016/j.optm.2008.10.015. Review. PubMed PMID:

19329062. **EXCLUDED, NOT INVESTIGATING/ESTIMATING OCCUPATIONAL EXPOSURE**

57: Lam LT. Uncorrected or untreated vision problems and occupational injuries

among the adolescent and adult population in Australia. Inj Prev. 2008

Dec;14(6):396-400. doi: 10.1136/ip.2008.018846. PubMed PMID: 19074246. **EXCLUDED, NOT INVESTIGATING/ESTIMATING OCCUPATIONAL EXPOSURE**

58: Lee L, Packer TL, Tang SH, Girdler S. Self-management education programs for

age-related macular degeneration: a systematic review. Australas J Ageing. 2008

Dec;27(4):170-6. doi: 10.1111/j.1741-6612.2008.00298.x. Review. PubMed PMID:

19032617. **EXCLUDED, REVIEW-COMMENT-LETTER**

59: Vukicevic M, Heriot W. Phototoxic maculopathy associated with arc welding:

clinical findings and associated functional vision impairment. Clin Exp

Ophthalmol. 2008 Oct;36(7):695-7. doi: 10.1111/j.1442-9071.2008.01864.x. PubMed

PMID: 18983562. **EXCLUDED, NOT SPECIFICALLY INVESTIGATING OUR OUTCOME**

60: Srisuwanporn S, Pattamasing na Ayutaya S, Wutthiphan S, Ratanasirintrawut S,

Sothornwit N, Jenchitr W, Pongprayoon C. Eye health in the priests and novices in

central Bangkok. J Med Assoc Thai. 2008;91 Suppl 1:S13-20. PubMed PMID: 18672585. **EXCLUDED, NOT SPECIFICALLY INVESTIGATING OUR OUTCOME**

61: Schexnaydre M, Carruth AK. My father's experience with macular degeneration:

implications for the home healthcare nurse. Home Healthc Nurse. 2008

Jan;26(1):8-14; quiz 15-6. Review. PubMed PMID: 18158486. **EXCLUDED, REVIEW-COMMENT-LETTER**

62: Eklund K, Dahlin-Ivanoff S. Low vision, ADL and hearing assistive device use

among older persons with visual impairments. Disabil Rehabil Assist Technol. 2007

Nov;2(6):326-34. PubMed PMID: 19263563. **EXCLUDED, NOT INVESTIGATING/ESTIMATING OCCUPATIONAL EXPOSURE**

63: Costa LG. Contaminants in fish: risk-benefit considerations. Arh Hig Rada

Toksikol. 2007 Sep;58(3):367-74. Review. PubMed PMID: 17913692. **EXCLUDED, REVIEW-COMMENT-LETTER**

64: Newsham Beckley M, Teaford MH, Kegelmeyer D, Balaswamy S, Flom R, Raasch T.

Interdisciplinary allied health education in treating older adults with low

vision. J Allied Health. 2007 Fall;36(3):e192-202. PubMed PMID: 19759992. **EXCLUDED, NOT INVESTIGATING/ESTIMATING OCCUPATIONAL EXPOSURE**

65: Eklund K, Sjöstrand J, Dahlin-Ivanoff S. A randomized controlled trial of a

health-promotion programme and its effect on ADL dependence and self-reported

health problems for the elderly visually impaired. Scand J Occup Ther. 2008

Jun;15(2):68-74. PubMed PMID: 17852958. **EXCLUDED, NOT INVESTIGATING/ESTIMATING OCCUPATIONAL EXPOSURE**

66: Adegbehingbe BO, Majengbasan TO. Ocular health status of rural dwellers in

south-western Nigeria. Aust J Rural Health. 2007 Aug;15(4):269-72. PubMed PMID:

17617092. **EXCLUDED, NOT INVESTIGATING/ESTIMATING OCCUPATIONAL EXPOSURE**

67: Kim EA, Kim BG, Yi CH, Kim IG, Chae CH, Kang SK. Macular degeneration in an

arc welder. Ind Health. 2007 Apr;45(2):371-3. PubMed PMID: 17485886. **EXCLUDED, CASE-REPORT**

68: Njirić S, Misljenović T, Mikulicić M, Pavicević L. Incidence of age related

macular degeneration in correlation with age, sex and occupation. Coll Antropol.

2007 Jan;31 Suppl 1:107-10. PubMed PMID: 17469763. **INCLUDED**

69: Peate WF. Work-related eye injuries and illnesses. Am Fam Physician. 2007 Apr

1;75(7):1017-22. Review. PubMed PMID: 17427615. **EXCLUDED, NOT SPECIFICALLY INVESTIGATING OUR OUTCOME**

70: Eklund K, Ivanoff SD. Health education for people with macular degeneration:

learning experiences and the effect on daily occupations. Can J Occup Ther. 2006

Dec;73(5):272-80. PubMed PMID: 17201100. **EXCLUDED, NOT INVESTIGATING/ESTIMATING OCCUPATIONAL EXPOSURE**

71: Shankar A, Mitchell P, Rochtchina E, Tan J, Wang JJ. Association between

circulating white blood cell count and long-term incidence of age-related macular

degeneration: the Blue Mountains Eye Study. Am J Epidemiol. 2007 Feb

15;165(4):375-82. PubMed PMID: 17110636. **EXCLUDED, NOT INVESTIGATING/ESTIMATING OCCUPATIONAL EXPOSURE**

72: Malek G, Cousins SW. Is our current clinical classification of AMD up to the

job? Br J Ophthalmol. 2006 Sep;90(9):1080-1. PubMed PMID: 16929056; PubMed

Central PMCID: PMC1857396. **EXCLUDED, REVIEW-LETTER-COMMENT**

73: Dantzig PI. Parkinson's disease, macular degeneration and cutaneous signs of

mercury toxicity. J Occup Environ Med. 2006 Jul;48(7):656. PubMed PMID: 16832218. **EXCLUDED, NOT INVESTIGATING/ESTIMATING OCCUPATIONAL EXPOSURE**

74: Lyons JS. Non-familial occult macular dystrophy. Doc Ophthalmol. 2005

Jul;111(1):49-56. PubMed PMID: 16502307. **EXCLUDED, NOT INVESTIGATING/ESTIMATING OCCUPATIONAL EXPOSURE**

75: Cohen SY, Legargasson JF. [Adaptation to central scotoma. Part II. Perceptual

filling-in phenomenon]. J Fr Ophtalmol. 2005 Dec;28(10):1131-6. Review. French.

PubMed PMID: 16395209. **EXCLUDED, NOT IN ENGLISH LANGUAGE**

76: Dahlin Ivanoff S, Sonn U. Assistive devices in activities of daily living

used by persons with age-related macular degeneration: a population study of

85-year-olds living at home. Scand J Occup Ther. 2005 Mar;12(1):10-7. PubMed

PMID: 16389994. **EXCLUDED, NOT INVESTIGATING/ESTIMATING OCCUPATIONAL EXPOSURE**

77: Nishiwaki Y, Tanaka E, Hirakata A, Oda K, Kigasawa K, Hida T. [A case report

illustrating the effectiveness of reading performance assessments in the

diagnosis and treatment of psychogenic visual disturbances]. Nippon Ganka Gakkai

Zasshi. 2005 Nov;109(11):761-5. Japanese. PubMed PMID: 16363671. **EXCLUDED, NOT IN ENGLISH LANGUAGE**

78: Eklund K, Sonn U, Nystedt P, Dahlin-Ivanoff S. A cost-effectiveness analysis

of a health education programme for elderly persons with age-related macular

degeneration: a longitudinal study. Disabil Rehabil. 2005 Oct 30;27(20):1203-12.

PubMed PMID: 16298922. **EXCLUDED, NOT INVESTIGATING/ESTIMATING OCCUPATIONAL EXPOSURE**

79: Saw SM, Gazzard G, Shih-Yen EC, Chua WH. Myopia and associated pathological

complications. Ophthalmic Physiol Opt. 2005 Sep;25(5):381-91. Review. PubMed

PMID: 16101943. **EXCLUDED, REVIEW-COMMENT-LETTER**

80: Maier R, Heilig P, Winker R, Neudorfer B, Hoeranter R, Ruediger H. Welder's

maculopathy? Int Arch Occup Environ Health. 2005 Sep;78(8):681-5. PubMed PMID:

16021465. **EXCLUDED, REVIEW-COMMENT-LETTER**

81: Nirmalan PK, Tielsch JM, Katz J, Thulasiraj RD, Krishnadas R, Ramakrishnan R,

Robin AL. Relationship between vision impairment and eye disease to

vision-specific quality of life and function in rural India: the Aravind

Comprehensive Eye Survey. Invest Ophthalmol Vis Sci. 2005 Jul;46(7):2308-12.

PubMed PMID: 15980215. **EXCLUDED, NOT INVESTIGATING/ESTIMATING OCCUPATIONAL EXPOSURE**

82: Yamagishi S, Nakamura K, Inoue H, Takeuchi M. Met72Thr polymorphism of

pigment epithelium-derived factor gene and susceptibility to age-related macular

degeneration. Med Hypotheses. 2005;64(6):1202-4. PubMed PMID: 15823717. **EXCLUDED, NOT INVESTIGATING/ESTIMATING OCCUPATIONAL EXPOSURE**

83: Murthy GV, Gupta SK, Thulasiraj RD, Viswanath K, Donoghue EM, Fletcher AE.

The development of the Indian vision function questionnaire: questionnaire

content. Br J Ophthalmol. 2005 Apr;89(4):498-503. PubMed PMID: 15774932; PubMed

Central PMCID: PMC1772602. **EXCLUDED, NOT INVESTIGATING/ESTIMATING OCCUPATIONAL EXPOSURE**

84: Nirmalan PK, Katz J, Robin AL, Ramakrishnan R, Krishnadas R, Thulasiraj RD,

Tielsch JM. Female reproductive factors and eye disease in a rural South Indian

population: the Aravind Comprehensive Eye Survey. Invest Ophthalmol Vis Sci. 2004

Dec;45(12):4273-6. PubMed PMID: 15557432. **EXCLUDED, NOT INVESTIGATING/ESTIMATING OCCUPATIONAL EXPOSURE**

85: Eklund K, Sonn U, Dahlin-Ivanoff S. Long-term evaluation of a health

education programme for elderly persons with visual impairment. A randomized

study. Disabil Rehabil. 2004 Apr 8;26(7):401-9. PubMed PMID: 15204476. **EXCLUDED, NOT INVESTIGATING/ESTIMATING OCCUPATIONAL EXPOSURE**

86: Browne M. The nurse's role in helping patients cope with sight loss. Nurs

Times. 2003 Dec 2-8;99(48):30-2. Review. PubMed PMID: 14705546. **EXCLUDED, REVIEW-COMMENT-LETTER**

87: Saw SM, Husain R, Gazzard GM, Koh D, Widjaja D, Tan DT. Causes of low vision

and blindness in rural Indonesia. Br J Ophthalmol. 2003 Sep;87(9):1075-8. PubMed

PMID: 12928268; PubMed Central PMCID: PMC1771857. **EXCLUDED, NOT INVESTIGATING/ESTIMATING OCCUPATIONAL EXPOSURE**

88: Koenekoop RK. The gene for Stargardt disease, ABCA4, is a major retinal gene:

a mini-review. Ophthalmic Genet. 2003 Jun;24(2):75-80. Review. Erratum in:

Ophthalmic Genet. 2003 Dec;24(4):253. PubMed PMID: 12789571. **EXCLUDED, REVIEW-COMMENT-LETTER**

89: Remé CE, Grimm C, Hafezi F, Iseli HP, Wenzel A. Why study rod cell death in

retinal degenerations and how? Doc Ophthalmol. 2003 Jan;106(1):25-9. Review.

PubMed PMID: 12675482. **EXCLUDED, REVIEW-COMMENT-LETTER**

90: Jobling AI, Fang Z, Koleski D, Tymms MJ. Expression of the ETS transcription

factor ELF3 in the retinal pigment epithelium. Invest Ophthalmol Vis Sci. 2002

Nov;43(11):3530-7. PubMed PMID: 12407165. **EXCLUDED, NOT INVESTIGATING/ESTIMATING OCCUPATIONAL EXPOSURE**

91: Dahlin Ivanoff S, Sonn U, Svensson E. A health education program for elderly

persons with visual impairments and perceived security in the performance of

daily occupations: a randomized study. Am J Occup Ther. 2002

May-Jun;56(3):322-30. PubMed PMID: 12058521. **EXCLUDED, NOT INVESTIGATING/ESTIMATING OCCUPATIONAL EXPOSURE**

92: Klein R, Klein BE, Jensen SC, Moss SE. The relation of socioeconomic factors

to the incidence of early age-related maculopathy: the Beaver Dam eye study. Am J

Ophthalmol. 2001 Jul;132(1):128-31. PubMed PMID: 11438075. **INCLUDED**

93: Rich D, Lane AM, Miller JW. Photodynamic therapy: the nurse's role. Insight.

2001 Apr;26(2):44-8. PubMed PMID: 11426204. **EXCLUDED, REVIEW-COMMENT-LETTER**

94: McCabe P, Nason F, Demers Turco P, Friedman D, Seddon JM. Evaluating the

effectiveness of a vision rehabilitation intervention using an objective and

subjective measure of functional performance. Ophthalmic Epidemiol. 2000

Dec;7(4):259-70. PubMed PMID: 11262673. **EXCLUDED, NOT INVESTIGATING/ESTIMATING OCCUPATIONAL EXPOSURE**

95: Nwosu SN. Low vision in Nigerians with diabetes mellitus. Doc Ophthalmol.

2000 Jul;101(1):51-7. PubMed PMID: 11128968. **EXCLUDED, NOT INVESTIGATING/ESTIMATING OCCUPATIONAL EXPOSURE**

96: Olson JA. Carotenoids and human health. Arch Latinoam Nutr. 1999 Sep;49(3

Suppl 1):7S-11S. Review. PubMed PMID: 10971837. **EXCLUDED, NOT INVESTIGATING/ESTIMATING OCCUPATIONAL EXPOSURE**

97: Khoo J, Saw SM, Banerjee K, Chia SE, Tan D. Outdoor work and the risk of

pterygia: a case-control study. Int Ophthalmol. 1998;22(5):293-8. PubMed PMID:

10826547. **EXCLUDED, NOT SPECIFICALLY INVESTIGATING OUR OUTCOME**

98: Blümel C, Brosig J. [Maculopathy caused by Nd:YAG laser accident]. Klin Monbl

Augenheilkd. 1999 Feb;214(2):112-4; discussion 114-5. German. PubMed PMID:

10218206. **EXCLUDED, NOT IN ENGLISH LANGUAGE**

99: Barton W. Role of ophthalmic nurses with visually impaired patients. Insight.

1998 Mar;23(1):5-10. PubMed PMID: 9866521. **EXCLUDED, REVIEW-LETTER-COMMENT**

100: Klein R, Cruickshanks KJ, Klein BE, Nondahl DM, Wiley T. Is age-related

maculopathy related to hearing loss? Arch Ophthalmol. 1998 Mar;116(3):360-5.

PubMed PMID: 9514490. **EXCLUDED, NOT INVESTIGATING/ESTIMATING OCCUPATIONAL EXPOSURE**

101: Attebo K, Mitchell P, Cumming R, Smith W. Knowledge and beliefs about common

eye diseases. Aust N Z J Ophthalmol. 1997 Nov;25(4):283-7. PubMed PMID: 9395831. **EXCLUDED, REVIEW-LETTER-COMMENT**

102: Richbourg MJ. Vision screening in older adults on dialysis: do nephrology

nurses have a role? ANNA J. 1997 Oct;24(5):541-4, 549, 555. PubMed PMID: 9392737. **EXCLUDED, REVIEW-LETTER-COMMENT**

103: Bressler NM, Munoz B, Maguire MG, Vitale SE, Schein OD, Taylor HR, West SK.

Five-year incidence and disappearance of drusen and retinal pigment epithelial

abnormalities. Waterman study. Arch Ophthalmol. 1995 Mar;113(3):301-8. PubMed

PMID: 7534060. **INCLUDED**

104: Swagerty DL Jr. The impact of age-related visual impairment on functional

independence in the elderly. Kans Med. 1995 Spring;96(1):24-6. PubMed PMID:

7666637. **EXCLUDED, NOT INVESTIGATING/ESTIMATING OCCUPATIONAL EXPOSURE**

105: Singh MC, Murthy GV, Venkatraman R, Nayar S. Epidemiological aspects of

visual impairment above 50 years in a rural area. J Indian Med Assoc. 1994

Nov;92(11):361-3, 365. PubMed PMID: 7890940. **EXCLUDED, NOT SPECIFICALLY INVESTIGATING OUR OUTCOME**

106: Buning ME, Hanzlik JR. Adaptive computer use for a person with visual

impairment. Am J Occup Ther. 1993 Nov;47(11):998-1008. PubMed PMID: 8279507. **EXCLUDED, NOT INVESTIGATING/ESTIMATING OCCUPATIONAL EXPOSURE**

107: Shiraishi N, Taguchi T, Kinebuchi H. Effect of age and sex on copper-induced

toxicity in the macular mutant mouse. An animal model for Menkes' kinky-hair

disease. Biol Trace Elem Res. 1993 Nov-Dec;39(2-3):129-37. PubMed PMID: 7509170.

**EXCLUDED, STUDY IN ANIMALS**

108: Phillips PM, Dean FM, Reffin J, Briggs TP, Miller RA, Davey CC. Does too

much urology damage your eyesight? Study of macular function. Br J Urol. 1992

Nov;70(5):488-91. PubMed PMID: 1467850. **EXCLUDED, NOT SPECIFICALLY INVESTIGATING OUR OUTCOME**

109: Wu ZQ. [Epidemiologic survey of senile macular degeneration]. Zhonghua Yan

Ke Za Zhi. 1992 Jul;28(4):246-7. Chinese. PubMed PMID: 1299574. **EXCLUDED, NOT IN ENGLISH LANGUAGE**

110: Bothe N, Hetzer R. [Etiology and occupational rehabilitation of late vision

disorders]. Klin Monbl Augenheilkd. 1992 Mar;200(3):237-41. German. PubMed PMID:

1578884. **EXCLUDED, NOT IN ENGLISH LANGUAGE**

111: Taylor HR, West S, Muñoz B, Rosenthal FS, Bressler SB, Bressler NM. The

long-term effects of visible light on the eye. Arch Ophthalmol. 1992

Jan;110(1):99-104. PubMed PMID: 1731731. **EXCLUDED REVIEW-LETTER-COMMENT**

112: Kordalewska A, Makszewska-Chetnikowa Z, Trusiewicz D, Zebrowska K.

[Ophthalmologic problems and occupational fitness]. Klin Oczna. 1989

Feb-Mar;91(2-3):41-2. Polish. PubMed PMID: 2593590. **EXCLUDED, NOT IN ENGLISH LANGUAGE**

113: Taylor HR. Ultraviolet radiation and the eye: an epidemiologic study. Trans

Am Ophthalmol Soc. 1989;87:802-53. PubMed PMID: 2562534; PubMed Central PMCID:

PMC1298564. **EXCLUDED, NOT SPECIFICALLY INVESTIGATING OUR OUTCOME**

114: Krawczykowa Z, Goś R, Orzeł H, Czajkowski J, Trzciński J, Stepień J, Pleszak

J, Ekman Z, Jarmak A, Berner B, et al. [Effects of petroleum derivatives and

psychological stress on eye changes in prospective studies]. Med Pr.

1988;39(3):212-6. Polish. PubMed PMID: 3226290. **EXCLUDED, NOT IN ENGLISH LANGUAGE**

115: Sanderson GF, Cumming AE, Polkinghorne PJ. A hospital rental system for low

vision aids. Aust N Z J Ophthalmol. 1986 Nov;14(4):359-63. PubMed PMID: 3814424. **EXCLUDED, NOT INVESTIGATING/ESTIMATING OCCUPATIONAL EXPOSURE**

116: Krawczykowa Z, Goś R, Orzeł H, Czajkowski J, Trzciński J, Stepień J, Pleszak

J, Berner B, Okraszewska E, Ekman Z, et al. [The state of the organ of vision in

workers in the petrochemical industry. I. Results of ophthalmological

examinations]. Med Pr. 1984;35(1):51-5. Polish. PubMed PMID: 6738350. **EXCLUDED, NOT IN ENGLISH LANGUAGE**

117: Clayton RM, Cuthbert J, Seth J, Phillips CI, Bartholomew RS, Reid JM.

Epidemiological and other studies in the assessment of factors contributing to

cataractogenesis. Ciba Found Symp. 1984;106:25-47. PubMed PMID: 6568978. **EXCLUDED, NOT SPECIFICALLY INVESTIGATING OUR OUTCOME**

118: Krawczykowa Z, Goś R, Orzeł H, Czajkowski J, Trzciński J, Stepień J, Pleszak

J, Berner B, Okraszewska E, Ekman Z, et al. [Evaluation of the organ of sight in

workers at the Masovian Refinery-Petrochemistry Plant. II. Correlation of the

results of ophthalmological tests with the results of specialized and laboratory

tests]. Med Pr. 1984;35(3):227-30. Polish. PubMed PMID: 6503691. **EXCLUDED, NOT IN ENGLISH LANGUAGE**

119: Goś R, Stepień J, Horowski P. [State of the eyes in welders of Division M-5,

Brown Coal Mine in Bełchatow]. Med Pr. 1984;35(2):133-6. Polish. PubMed PMID:

6472095. **EXCLUDED, NOT IN ENGLISH LANGUAGE**

120: Hyman LG, Lilienfeld AM, Ferris FL 3rd, Fine SL. Senile macular

degeneration: a case-control study. Am J Epidemiol. 1983 Aug;118(2):213-27.

PubMed PMID: 6881127. **INCLUDED**

121: Hartmann E, Scheffzyk-Hagl A, Lachenmayr B. [Influence of luminous

intensity, contrast and color on vision in patients with slight to severe visual

defects (author's transl)]. Klin Monbl Augenheilkd. 1980 Sep;177(3):304-18.

German. PubMed PMID: 7453066. **EXCLUDED, NOT IN ENGLISH LANGUAGE**

122: Pambor R, Giessmann HG, Kielstein R, Lachhein L. [Eye fundus pathology in

chronic terminal kidney failure]. Z Gesamte Inn Med. 1979 Apr 1;34(7):205-8.

German. PubMed PMID: 483928. **EXCLUDED, NOT IN ENGLISH LANGUAGE**

123: Kornzweig AL. A low-vision clinic at a home for the aged. J Am Geriatr Soc.

1976 Dec;24(12):538-41. PubMed PMID: 993545. **EXCLUDED, NOT INVESTIGATING/ESTIMATING OCCUPATIONAL EXPOSURE**

124: Fülle HH. [Incidence, age and occupational distribution of senile and myopic

macular degeneration]. Klin Monbl Augenheilkd. 1968;153(4):578-88. German. PubMed

PMID: 5714770. **EXCLUDED, NOT IN ENGLISH LANGUAGE**

**Other SCOPUS results after the removal of 94 PubMed duplicates from a total of 128 hits**

**- Scientific Literature Analyzed from 1st March 1967 to 1st March 2017**

1. Kyari, F., Wormald, R., Murthy, G.V.S., Evans, J.R., Gilbert, C.E. Ethnicity and deprivation are associated with blindness among adults with primary glaucoma in Nigeria: Results from the Nigeria National Blindness and Visual Impairment Survey (2016) Journal of Glaucoma, 25 (10), pp. e861-e872. https://www.scopus.com/inward/record.uri?eid=2-s2.0 84980329389&doi=10.1097%2fIJG.0000000000000487&partnerID=40&md5=4867acef3256579 1bb326b3e87c28b1 DOI: 10.1097/IJG.0000000000000487 DOCUMENT TYPE: Conference Paper

**EXCLUDED, NOT SPECIFICALLY INVESTIGATING OUR OUTCOME**

2. Kasi, S.K., Regillo, C.D. A team-based approach to treating advanced macular degeneration: The combination of candidate selection, occupational therapy, and implantable miniature telescope surgery is not a one-person job (2016) Retina Today, 2016 (September), pp. 64-66. https://www.scopus.com/inward/record.uri?eid=2-s2.0 84987991110&partnerID=40&md5=7ee5c450eb519c5b55c32ce8d2d6463c DOCUMENT TYPE: Note

**EXCLUDED, NOT INVESTIGATING/ESTIMATING OCCUPATIONAL EXPOSURE**

3. Cimarolli, V.R., Casten, R.J., Rovner, B.W., Heyl, V., Sörensen, S., Horowitz, A. Anxiety and depression in patients with advanced macular degeneration: Current perspectives (2016) Clinical Ophthalmology, 10, pp. 55-63. Cited 3 times. https://www.scopus.com/inward/record.uri?eid=2-s2.0 84954437811&doi=10.2147%2fOPTH.S80489&partnerID=40&md5=d7e06ea900c1e6346814538a 7c6ab01 DOI: 10.2147/OPTH.S80489 DOCUMENT TYPE: Review **EXCLUDED-REVIEW.LETTER-COMMENT**

4. Kennedy, R.D., Douglas, O. Strategies to help patients stop smoking: The optometrist’s perspective (2015) Clinical Optometry, 7, pp. 103-113. https://www.scopus.com/inward/record.uri?eid=2-s2.0 84981709055&doi=10.2147%2fOPTO.S63185&partnerID=40&md5=475abe3fc934ed230e36775e 1d90948 DOI: 10.2147/OPTO.S63185 DOCUMENT TYPE: Review **EXCLUDED-REVIEW.LETTER-COMMENT**

5. Wright, F., Weller, R.B. Risks and benefits of UV radiation in older people: More of a friend than a foe?(2015) Maturitas, 81 (4), pp. 425-431. Cited 3 times. https://www.scopus.com/inward/record.uri?eid=2-s2.0-84938201530&doi=10.1016%2fj.maturitas.2015.05.003&partnerID=40&md5=1637c2beb3f4d7616a0b58321c8e2e85 DOI: 10.1016/j.maturitas.2015.05.003 DOCUMENT TYPE: Review **EXCLUDED-REVIEW.LETTER-COMMENT**

6. Stevens, R., Bartlett, H., Cooke, R. Dietary analysis and nutritional behavior in people with and without age-related macular disease(2015) Clinical Nutrition ESPEN, 10 (3), pp. e112-e117. https://www.scopus.com/inward/record.uri?eid=2 s2.0-84957806147&doi=10.1016%2fj.clnesp.2015.03.080&partnerID=40&md5=597c6bca42fc85dab5b055bca4b4059c DOI: 10.1016/j.clnesp.2015.03.080 DOCUMENT TYPE: Article **EXCLUDED, NOT INVESTIGATING/ESTIMATING OCCUPATIONAL EXPOSURE**

7. Trevillian, S., Marks, M.

An interview with maurice marks

(2015) Journal of Occupational Science, 22 (2), pp. 242-245. Cited 1 time.

https://www.scopus.com/inward/record.uri?eid=2-s2.0-84961360717&doi=10.1080%2f14427591.2013.775637&partnerID=40&md5=4bf985940ba6be1ed1e14c5fc777d9c1

DOI: 10.1080/14427591.2013.775637

DOCUMENT TYPE: Review

SOURCE: Scopus **EXCLUDED-REVIEW-LETTER-COMMENT**

8. Morgan, S.

General practice: Font and centre

(2015) British Journal of General Practice, 65 (638), p. 478.

https://www.scopus.com/inward/record.uri?eid=2-s2.0-84942567420&doi=10.3399%2fbjgp15X686617&partnerID=40&md5=31fad458f8dc8fb82e1d6893730fb78c

DOI: 10.3399/bjgp15X686617

DOCUMENT TYPE: Note

SOURCE: Scopus **EXCLUDED, NOT INVESTIGATING/ESTIMATING OCCUPATIONAL EXPOSURE**

9. Panesar, K.

Age-related macular degeneration

(2015) U.S. Pharmacist, 40 (6), pp. 22-26.

https://www.scopus.com/inward/record.uri?eid=2-s2.0-84932615281&partnerID=40&md5=af7b2176581f44d9addf0d53a218469d

DOCUMENT TYPE: Article

SOURCE: Scopus **EXCLUDED, NOT INVESTIGATING/ESTIMATING OCCUPATIONAL EXPOSURE**

10. Copolillo, A.

Teaching/learning strategies for intervention with people with neurovisual impairments

(2015) International Handbook of Occupational Therapy Interventions, Second Edition, pp. 487-496.

https://www.scopus.com/inward/record.uri?eid=2-s2.0-84944753149&doi=10.1007%2f978-3-319-08141-0_32&partnerID=40&md5=5722f58cb4794d1cc554d219e0e6a15d **EXCLUDED, NOT INVESTIGATING/ESTIMATING OCCUPATIONAL EXPOSURE**

DOI: 10.1007/978-3-319-08141-0_32

DOCUMENT TYPE: Book Chapter

SOURCE: Scopus

11. Omoso, M.R., Gloria, A.E.

Pattern and prevalence of eye diseases among farmers Inan agricultural industry in southern Nigeria

(2015) Journal of Medicine and Biomedical Research, 14 (2), pp. 73-80.

https://www.scopus.com/inward/record.uri?eid=2-s2.0-84988959008&partnerID=40&md5=906c2668534fecf73a314c9c776ad5c9

DOCUMENT TYPE: Article

SOURCE: Scopus

**EXCLUDED, NOT SPECIFICALLY INVESTIGATING OUR OUTCOME**

12. Cardarelli, W.J., Smith, R.A.

Managed care implications of age-related ocular conditions

(2013) American Journal of Managed Care, 19 (5 SUPPL.), . Cited 8 times.

https://www.scopus.com/inward/record.uri?eid=2-s2.0-84894424003&partnerID=40&md5=2507fa48b8204b69b5587819365f323b

DOCUMENT TYPE: Review

SOURCE: Scopus

**EXCLUDED, REVIEW-LETTER-COMMENT**

13. You, Q.S., Xu, L., Yang, H., Li, Y.B., Wang, S., Wang, J.D., Zhang,J.S., Wang, Y.X., Jonas, J.B

Five-year incidence of age-related macular degeneration: The Beijing eye study

(2012) Ophthalmology, 119 (12), pp. 2519-2525. Cited 26 times.

https://www.scopus.com/inward/record.uri?eid=2-s2.0-84870653405&doi=10.1016%2fj.ophtha.2012.06.043&partnerID=40&md5=999a4fcb3efaf4f700f5fb839eafdeda

DOI: 10.1016/j.ophtha.2012.06.043

DOCUMENT TYPE: Article

SOURCE: Scopus **EXCLUDED, NOT INVESTIGATING/ESTIMATING OCCUPATIONAL EXPOSURE**

14. El Chehab, H., Blein, J.-P., Herry, J.-P., Chave, N., Ract-Madoux, G., Agard, E., Guarracino, G., Swalduz, B., Mourgues, G., Dot, C.

Ocular phototoxicity and altitude among mountain guides [Phototoxicité oculaire et altitude chez des guides de haute montagne]

(2012) Journal Francais d'Ophtalmologie, 35 (10), pp. 809-815. Cited 2 times.

https://www.scopus.com/inward/record.uri?eid=2-s2.0-84870948361&doi=10.1016%2fj.jfo.2012.06.012&partnerID=40&md5=b0b4fbb200c56caa00dbe18fb1bb091b

DOI: 10.1016/j.jfo.2012.06.012

DOCUMENT TYPE: Article

SOURCE: Scopus **EXCLUDED, NOT IN ENGLISH LANGUAGE**

15. Steinberg, J., Uibel, S., Berndt, T., Müller, D., Quarcoo, D., Groneberg, D.A.

Age-related macular degeneration [Die altersabhängige makuladegeneration -eine übersicht]

(2011) Zentralblatt fur Arbeitsmedizin, Arbeitsschutz und Ergonomie, 61(8), pp. 270-286.

https://www.scopus.com/inward/record.uri?eid=2-s2.0-79961200423&partnerID=40&md5=126c54b2ab1cab33371db4552d707d70 **EXCLUDED, NOT IN ENGLISH LANGUAGE**

DOCUMENT TYPE: Article

SOURCE: Scopus

16. Lucas, R.M.

An epidemiological perspective of ultraviolet exposure-public health concerns

(2011) Eye and Contact Lens, 37 (4), pp. 168-175. Cited 22 times.

https://www.scopus.com/inward/record.uri?eid=2-s2.0-79959982264&doi=10.1097%2fICL.0b013e31821cb0cf&partnerID=40&md5=fb296e45d88456b3a3b2450536bdd55a

DOI: 10.1097/ICL.0b013e31821cb0cf

DOCUMENT TYPE: Review

SOURCE: Scopus

**EXCLUDED, REVIEW-COMMENT-LETTER**

17. Golka, K., Selinski, S., Lehmann, M.-L., Blaszkewicz, M., Marchan, R., Ickstadt, K., Schwender, H., Bolt, H.M., Hengstler, J.G.

Genetic variants in urinary bladder cancer: Collective power of the "wimp SNPs"

(2011) Archives of Toxicology, 85 (6), pp. 539-554. Cited 45 times.

https://www.scopus.com/inward/record.uri?eid=2-s2.0-79959612766&doi=10.1007%2fs00204-011-0676-3&partnerID=40&md5=c946d14cf695e1ebcab8ca293c06f1ee

DOI: 10.1007/s00204-011-0676-3

DOCUMENT TYPE: Review

SOURCE: Scopus **EXCLUDED, REVIEW-COMMENT-LETTER**

18. Dugel, P.U., Tong, K.B.

Development of an activity-based costing model to evaluate physician office practice profitability

(2011) Ophthalmology, 118 (1), pp. 203-208. Cited 5 times.

https://www.scopus.com/inward/record.uri?eid=2-s2.0-78650814436&doi=10.1016%2fj.ophtha.2010.04.035&partnerID=40&md5=24797d2cd4a710c149334fcd88936f84

DOI: 10.1016/j.ophtha.2010.04.035

DOCUMENT TYPE: Article

SOURCE: Scopus **EXCLUDED, NOT SOECIFICALLY INVESTIGATING OUR OUTCOME**

19. Ravin, J.G.

The visual difficulties of selected artists and limitations of ophthalmological care during the 19th and early 20th centuries (An AOS thesis)

(2008) Transactions of the American Ophthalmological Society, 106, pp. 402-425. Cited 2 times.

https://www.scopus.com/inward/record.uri?eid=2-s2.0-63049132476&partnerID=40&md5=f5060794105e8dbe3245834805d2561a

DOCUMENT TYPE: Article

SOURCE: Scopus

**EXCLUDED, NOT SPECIFICALLY INVESTIGATING OUR OUTCOME**

20. Teichman, J.C., Markowitz, S.N.

Canadian research contributions to low-vision rehabilitation

(2008) Canadian Journal of Ophthalmology, 43 (4), pp. 414-418. Cited 5 times.

https://www.scopus.com/inward/record.uri?eid=2-s2.0-49949107873&doi=10.3129%2fI08-065&partnerID=40&md5=a3f5e6aab089aaa85c336a8f3f7957a5

DOI: 10.3129/I08-065

DOCUMENT TYPE: Article

SOURCE: Scopus

**EXCLUDED, NOT INVESTIGATING/ESTIMATING OCCUPATIONAL EXPOSURE**

21. Casten, R.J., Maloney, E.K., Rovner, B.W.

Knowledge and use of low vision services among persons with age-related macular degeneration

(2005) Journal of Visual Impairment and Blindness, 99 (11), pp. 720-724. Cited 4 times.

https://www.scopus.com/inward/record.uri?eid=2-s2.0-33846705780&partnerID=40&md5=72ae48f1ac88d0731d74e8cbb042e515

DOCUMENT TYPE: Article

SOURCE: Scopus **EXCLUDED, NOT INVESTIGATING/ESTIMATING OCCUPATIONAL EXPOSURE**

22. Lisi, F.

The visually impaired and their social integration in the field of work

(2005) International Congress Series, 1282, pp. 1158-1162.

https://www.scopus.com/inward/record.uri?eid=2-s2.0-33646461149&doi=10.1016%2fj.ics.2005.06.001&partnerID=40&md5=09f5455a859be154743a865701f2dd6b

DOI: 10.1016/j.ics.2005.06.001

DOCUMENT TYPE: Article

SOURCE: Scopus

**EXCLUDED, NOT INVESTIGATING/ESTIMATING OCCUPATIONAL EXPOSURE**

23. Nirmalan, P.K., Katz, J., Tielsch, J.M., Robin, A.L., Thulasiraj, R.D., Krishnadas, R., Ramakrishnan, R.

Ocular trauma in a rural south Indian population: The AravindComprehensive Eye Survey

(2004) Ophthalmology, 111 (9), pp. 1778-1781. Cited 37 times.

https://www.scopus.com/inward/record.uri?eid=2-s2.0-4444344580&doi=10.1016%2fj.ophtha.2004.02.012&partnerID=40&md5=b702b4a4b6e1d4cd4114586c72d9defd

DOI: 10.1016/j.ophtha.2004.02.012

DOCUMENT TYPE: Review

SOURCE: Scopus

**EXCLUDED, NOT SPECIFICALLY INVESTIGATING OUR OUTCOME**

24. Kocur, I., Resnikoff, S.

Visual impairment and blindness in Europe and their prevention

(2002) British Journal of Ophthalmology, 86 (7), pp. 716-722. Cited 120 times.

https://www.scopus.com/inward/record.uri?eid=2-s2.0-0036291931&doi=10.1136%2fbjo.86.7.716&partnerID=40&md5=880977282e8f5a68f5ee66c6f757b867

DOI: 10.1136/bjo.86.7.716

DOCUMENT TYPE: Review

SOURCE: Scopus

**EXCLUDED, REVIEW-COMMENT-LETTER**

25. Dahlin Ivanoff, S.

Focus group discussions as a tool for developing a health education programme for elderly persons with visual impairment

(2002) Scandinavian Journal of Occupational Therapy, 9 (1), pp. 3-9. Cited 33 times.

https://www.scopus.com/inward/record.uri?eid=2-s2.0-0036072774&doi=10.1080%2f110381202753505809&partnerID=40&md5=726456eb3b3b54f3e5e1befb0f4ae2e8

DOI: 10.1080/110381202753505809

DOCUMENT TYPE: Article

SOURCE: Scopus

**EXCLUDED, NOT INVESTIGATING/ESTIMATING OCCUPATIONAL EXPOSURE**

26. Dahlin Ivanoff, S.

Development and evaluation of a health education programme for elderly persons with age-related macular degeneration

(2001) Scandinavian Journal of Occupational Therapy, 8 (2), p. 105. Cited 1 time.

https://www.scopus.com/inward/record.uri?eid=2-s2.0-0034963155&partnerID=40&md5=684296d866d11e6e6a14e57e3bf308a8

DOCUMENT TYPE: Article

SOURCE: Scopus

**EXCLUDED, NOT INVESTIGATING/ESTIMATING OCCUPATIONAL EXPOSURE**

27. Maino, J.H.

Visual deficits and mobility: Evaluation and management

(1996) Clinics in Geriatric Medicine, 12 (4), pp. 803-823. Cited 9 times.

https://www.scopus.com/inward/record.uri?eid=2-s2.0-0029829484&partnerID=40&md5=f89b5c902c43d39633527adba98b53f9

DOCUMENT TYPE: Review

SOURCE: Scopus

**EXCLUDED, REVIEW-LETTER-COMMENT**

28. Smith, A.J., Aston, S.J.

The use of activity in rehabilitation of elders with vision impairments

(1989) Topics in Geriatric Rehabilitation, 4 (4), pp. 45-52.

https://www.scopus.com/inward/record.uri?eid=2-s2.0-0024374510&partnerID=40&md5=05f0f58a7593a5ff383244bde8c7bdad

DOCUMENT TYPE: Article

SOURCE: Scopus

**EXCLUDED, NOT INVESTIGATING/ESTIMATING OCCUPATIONAL EXPOSURE**

29. Weinstein, G.W.

Ophthalmology in service to the public

(1985) Archives of Ophthalmology, 103 (3), pp. 345-346. Cited 1 time.

https://www.scopus.com/inward/record.uri?eid=2-s2.0-0021947996&doi=10.1001%2farchopht.1985.01050030041016&partnerID=40&md5=2bb1a0178fe20d1f7e8a82090cc94f26

DOI: 10.1001/archopht.1985.01050030041016

DOCUMENT TYPE: Article

SOURCE: Scopus

**EXCLUDED, NOT INVESTIGATING/ESTIMATING OCCUPATIONAL EXPOSURE**

30. LOVIE‐KITCHIN, J.E., BOWMAN, K.J., FARMER, E.J., WELLS, S., SUTTIE, B., BURROUGH, S.

Senile Macular Degeneration – The Effects and Management: VI. The Visual Handicap

(1984) The Australian Journal of Optometry, 67 (4), pp. 118-125.

https://www.scopus.com/inward/record.uri?eid=2-s2.0-84990623535&doi=10.1111%2fj.1444-0938.1984.tb02366.x&partnerID=40&md5=12b050a11845f29420cab5b6d89c0af7

DOI: 10.1111/j.1444-0938.1984.tb02366.x

DOCUMENT TYPE: Article

SOURCE: Scopus

**EXCLUDED, NOT INVESTIGATING/ESTIMATING OCCUPATIONAL EXPOSURE**

31. Ham, W.T.

Ocular hazards of light sources: Review of current knowledge

(1983) Journal of Occupational Medicine, 25 (2), pp. 101-103. Cited 39 times.

https://www.scopus.com/inward/record.uri?eid=2-s2.0-0020582201&partnerID=40&md5=4abb56775953c0f18661853d3f085a8d

DOCUMENT TYPE: Article

SOURCE: Scopus

**EXCLUDED, REVIEW-LETTER-COMMENT**

32. Misra, U.K., Nag, D., Misra, N.K., Krishna Murti, C.R.

MACULAR DEGENERATION ASSOCIATED WITH CHRONIC PESTICIDE EXPOSURE

(1982) The Lancet, 319 (8266), p. 288. Cited 2 times.

https://www.scopus.com/inward/record.uri?eid=2-s2.0-0020047094&doi=10.1016%2fS0140-6736%2882%2991015-7&partnerID=40&md5=ce290cbb78ef771ca97c7180701d3150

DOI: 10.1016/S0140-6736(82)91015-7

DOCUMENT TYPE: Letter

SOURCE: Scopus

**EXCLUDED, REVIEW-LETTER-COMMENT**

33. Bruckner, R.

Professional rehabilitation of visually impaired patients [RUCKGLIEDERUNG SEHBEHINDERTER IN DEN FRUHEREN BERUF]

(1979) Schweizerische Rundschau fur Medizin/Praxis, 68 (50), pp. 1657-1665.

https://www.scopus.com/inward/record.uri?eid=2-s2.0-0018568894&partnerID=40&md5=4873df4b3f4afc4d1e6bba2680512b9a

DOCUMENT TYPE: Article

SOURCE: Scopus

**EXCLUDED, NOT IN ENGLISH LANGUAGE**

34. Pape, R.

Occupational rehabilitation of patients with lesions of the macula [DIE BERUFLICHE REHABILITATION VON PATIENTEN MIT MACULASCHADEN]

(1975) BER.DTSCH.OPHTHAL.GESELLSCH., vol.73, pp. 601-603.

https://www.scopus.com/inward/record.uri?eid=2-s2.0-0016771701&partnerID=40&md5=91ed60482a5f8b4a758b9f8bbe87fb10

DOCUMENT TYPE: Article

SOURCE: Scopus

**EXCLUDED, NOT IN ENGLISH LANGUAGE**
